# Promotion of metastasis by a specific complex of coagulation factors may be independent of fibrin formation.

**DOI:** 10.1038/bjc.1988.184

**Published:** 1988-08

**Authors:** P. McCulloch, W. D. George

**Affiliations:** University Department of Surgery, Western Infirmary, Glasgow, UK.

## Abstract

Coumarins inhibit metastasis in a number of animal models, but the mechanism of this effect remains unclear. We have investigated the relationship between the coagulation system and metastasis using a new model system, involving i.v. injection of Mtln3 rat mammary carcinoma cells into Fischer 344 rats, and subsequent estimation of pulmonary seeding. Injection of factors II, VII, IX and X elevated the median number of surface pulmonary seedlings per animal to 182, and injection of factors II, IX and X to 181, compared with a median for control animals of 12 (P less than 0.001). Injection of factor VII alone, or of bovine serum albumin did not significantly affect pulmonary seeding. In a second experiment, arvin defibrination reduced the mean plasma fibrinogen concentration to 76.8 mg dl-1 from a control value of 228 mg dl-1. This degree of defibrination had no significant effects on pulmonary seeding, nor on the enhancing effects of factor complex injection (median numbers of seedlings per animal; control 15, arvin 21, arvin plus factors II, VII, IX and X 170, factors II, VII, IX and X only, 157). Factor complex injections did not detectably shorten thrombotest clotting times. In vitro testing suggested that Mtln3 cells contain little or no conventional factor X activating cancer procoagulant. The complex of coagulation factors II, IX and X appears to contain a component which greatly enhances metastasis in this model. This may explain the previously reported antimetastatic effect of coumarin anticoagulants, which suppress factors II, VII, IX and X. The enhancing effect of the factor complex does not appear to be altered by significant reductions in fibrin forming capacity, and defibrination itself has no effect on metastasis. These findings suggest the possibility that the effect of this factor complex on metastasis may be mediated via mechanisms other than the formation of a fibrin clot.


*Br. J. Cancer* (1988), **58**, 158–162 © The Macmillan Press Ltd., 1988

### Promotion of metastasis by a specific complex of coagulation factors may be independent of fibrin formation

P. McCulloch & W.D. George

*University Department of Surgery, Western Infirmary, Glasgow, UK.*

**Summary** Coumarins inhibit metastasis in a number of animal models, but the mechanism of this effect remains unclear. We have investigated the relationship between the coagulation system and metastasis using a new model system, involving i.v. injection of Mtln3 rat mammary carcinoma cells into Fischer 344 rats, and subsequent estimation of pulmonary seeding.

Injection of factors II, VII, IX and X elevated the median number of surface pulmonary seedlings per animal to 182, and injection of factors II, IX and X to 181, compared with a median for control animals of 12 ($P<0.001$). Injection of factor VII alone, or of bovine serum albumin did not significantly affect pulmonary seeding. In a second experiment, arvin defibrination reduced the mean plasma fibrinogen concentration to 76.8 $mg\,dl^{-1}$ from a control value of 228 $mg\,dl^{-1}$. This degree of defibrination had no significant effects on pulmonary seeding, nor on the enhancing effects of factor complex injection (median numbers of seedlings per animal; control 15, arvin 21, arvin plus factors II, VII, IX and X 170, factors II, VII, IX and X only, 157). Factor complex injections did not detectably shorten thrombotest clotting times. *In vitro* testing suggested that Mtln3 cells contain little or no conventional factor X activating cancer procoagulant.

The complex of coagulation factors II, IX and X appears to contain a component which greatly enhances metastasis in this model. This may explain the previously reported antimetastatic effect of coumarin anticoagulants, which suppress factors II, VII, IX and X. The enhancing effect of the factor complex does not appear to be altered by significant reductions in fibrin forming capacity, and defibrination itself has no effect on metastasis. These findings suggest the possibility that the effect of this factor complex on metastasis may be mediated via mechanisms other than the formation of a fibrin clot.

There is extensive evidence from both clinical and experimental studies for an interaction between the coagulation system and the spread and growth of malignant disease (Wood, 1958; O'Meara, 1968; Hilgard *et al.*, 1977; Zacharski *et al.*, 1979; Dvorak *et al.*, 1981). Patient studies have demonstrated the existence of marked subclinical disturbances of the coagulation system in nearly all cancer patients (Sun *et al.*, 1979; Rickles & Edwards, 1983; Mannuci *et al.*, 1985), whilst animal experiments have suggested that the coagulation system may play an important role in the pathogenesis of blood borne metastasis (Koike, 1964; Agostino *et al.*, 1966; Brown, 1973; Wood, 1974; Poggi *et al.*, 1978). The most striking and consistent finding in such animal experimentation has been the antimetastatic effect of the coumarin group of anticoagulant drugs in a variety of tumour/host combinations (Ryan *et al.*, 1969; Hilgard & Maat, 1979; Williamson *et al.*, 1980). The coumarins mediate their anticoagulant activity by antagonising the action of vitamin K, an essential cofactor in the hepatic synthesis of the coagulation factors II (prothrombin), VII, IX and X (Stenflo & Suttie, 1977). We have demonstrated that warfarin, a member of the coumarin group, inhibits metastasis in a model system comprising the Mtln3 rat mammary carcinoma clone and the syngeneic Fischer 344 rat, both when intravenous injection of the cells is employed and in the more realistic model involving spontaneous metastasis (McCulloch & George, 1987). In addition, we showed that warfarin has no important cytotoxic effects for these tumour cells, that it inhibits metastasis principally by its effects on the host animal, and that the inhibition of metastasis is reversed by replenishment of the coagulation factors which warfarin suppresses. The exact role of these factors in the metastatic process therefore merited further study. The present studies were designed to determine whether the previously studied factor complex, or parts of it, could enhance metastasis in normal rats, and if so, whether this enhancing effect was dependent on a normal capacity to form fibrin.

Correspondence: W.D. George.
Received 17 December 1987; and in revised form, 15 March 1988.

### Animals and methods

### *Animals*

Female Fischer 344 rats (Olac Limited, Bicester, UK), 6–8 weeks old, mean weight 140 g, were used in all experiments. Animals were fed a standard laboratory diet (CRM diet, Labsure, Cambridge, UK) and tap water with a chlorine content of 7 $mg\,l^{-1}$. All animals were healthy according to visual observations, and to the results of routine microbiological testing for infection.

### *Tumour cells*

The tumour cells were a clone of rat mammary carcinoma designated Mtln3, originally derived by Neri and Nicolson (Neri *et al.*, 1982) from the 7,12-dimethylbenz(a)anthracene-induced adenocarcinoma 13762 (Segaloff, 1966). Cells were cultured in 75 $cm^2$ tissue culture flasks (Gibco, Paisley, UK) in equal parts of Hams' F10 and Dulbecco's modified Eagles' Medium (F10/DMEM), with 10% foetal calf serum (FCS) but without antibiotics. Cultures were maintained at 37°C in equilibrium with 2% $Co_2$ in air. Subconfluent cultures were passaged by the use of $Ca^{2+}$ and $Mg^{2+}$ free PBS followed by 0.25% Trypsin (Gibco, Paisley, UK). Subculture was performed by adding $3\times10^6$ viable cells to further 75 $cm^2$ flasks. Cells were passaged a maximum of six times between thawing and use, to minimise problems of phenotypic drift (Neri & Nicolson, 1981). Multiple subcultures of the cell line were stored in liquid nitrogen at −196°C, and fresh cultures were begun from these as required. Inocula of $10^6$ cells from stock cultures injected into the mammary fat pad of Fischer rats at the beginning and at the end of this series of experiments showed no change in the metastatic potential of the line.

### *Coagulation factor preparations*

A heat treated concentrate of human coagulation factors II, IX and X, prepared from pooled plasma by crypoprecipitation and supernatant adsorption with DEAE cellulose, was obtained from Dr R.J. Perry of the Protein Fractionation Centre, Edinburgh, UK. A heat treated concentrate of human factor VII, prepared by a similar procedure using DEAE sepharose, was obtained from Dr J.K. Smith of the Protein Fractionation Centre, Churchill Hospital, Oxford, UK. The factors were administered according to a regimen which had been shown in previous experiments to reconstitute coagulation in the fully warfarinised rat for ~12 h (McCulloch & George, 1987). Each rat was given a dose representing 6 units of factors II and X, 7 units of factor IX and 10 units of factor VII at the time of tumour cell injection, and the dose was repeated after 6 h. The fluid volume of the factor injections totalled 0.6 ml. One unit is sufficient to restore 1 ml of completely depleted human plasma to normal activity for the factor concerned.

*Experimental model of metastasis*

The model of metastasis used involved intravenous injection of Mtln3 tumour cells into F344 rats, with subsequent sacrifice and examination of the lungs for tumour seeding. Mtln3 cells were prepared from subconfluent cultures as described above, then washed twice by centrifugation for 5 min at 200 *g* in F10/DMEM with FCS, and a third time in F10/DMEM alone. After resuspension in F10/DMEM, cell density was assessed using a Coulter model ZB cell counter, and viability by Trypan Blue exclusion. All cultures used were >90% viable. An injection of 0.2 ml of this cell suspension was made into the lateral tail vein of F344 rats under light ether anaesthesia. Rats were maintained on normal diet and water for 17 days, then killed by cervical dislocation. Full autopsy was performed on all animals, and any tissue suspected of containing tumour deposits was submitted to histological examination. The lungs were prepared for assessment of pulmonary seeding by the method of Wexler (1966). This entails inflation of the lungs via the trachea with a 15% solution of india ink, followed by fixation in Fekete's solution for at least 48 h. Surface pulmonary tumour nodules can then be identified and counted accurately. All specimens were counted on two occasions by a single observer, who was unaware of the treatment given.

*Experiment 1: Enhancement of metastasis by coagulation factors*

Five groups of 10 F344 female rats, 6–8 weeks old, were used. All animals were injected intravenously with $10^4$ Mtln3 cells as described above. At the same time, additional treatments were begun as follows:

*Group A:* These control animals received no form of treatment other than tumour cell injection.

*Group B:* These animals received two i.v. injections of a complex of the coagulation factors II, VII, IX and X, as described above.

*Group C:* These animals received two i.v. injections of factor VII alone.

*Group D:* These animals received two i.v. injections of the complex of factors II, IX and X, but not factor VII.

*Group E:* These animals received two i.v. injections of bovine serum albumin (Sigma, Poole, UK).

No animal received treatment with warfarin or any other anticoagulant. In all groups receiving some form of treatment (Groups B–E) the first intravenous injection was given at the same time as the tumour cells, and the second 6 h later. The dose of bovine serum albumin used was 30 mg in 0.6 ml of F10/DMEM; this gave the same protein concentration as the factor complex injection in Group B rats. Bovine serum albumin and factor complex preparations were passed through a 0.2 $\mu$m filter before injection, for sterilization and removal of any potentially embolic material. Comparison of the numbers of seedlings per set of lungs in the different groups was performed by Mann–Whitney U test.

*Experiment 2: Role of fibrin formation in enhancement of metastasis*

Four groups of 10 Fischer 344 female rats, 6–8 weeks old, were used. All animals were injected i.v. with $10^4$ Mtln3 cells as described above. At the same time, additional treatments were begun as follows:

*Group A:* These control animals received no form of treatment other than tumour cell injection.

*Group B:* These animals received two i.v. injections of a complex of the factors II, VII, IX and X.

*Group C:* These animals received factor complex injections in the same way as group B animals, but received additional treatment before and at the time of tumour cell injection with arvin.

*Group D:* These animals received treatment with arvin according to the same protocol as group C animals, but did not receive any factor complex injections.

Factor complex injections were given as in experiment 1. Arvin was given i.v. in a dose of 150 units $kg^{-1}$, 6 h before, and s.c. in the same dose 6 h after injection of tumour cells. The arvin solution was filtered in the same way as the factor complex injections. Sacrifice, autopsy and estimation of pulmonary metastasis was performed exactly as in experiment 1.

*Monitoring of coagulation system activity*

The effects of the various treatments on coagulation were monitored by performing thrombotest (Nyegaard, Oslo, Norway) estimations on 3 animals per group immediately after the first injection. Tail vein blood (50 $\mu$l) was used for the assay. In experiment 2 the effects of arvin treatment were monitored by measurement of plasma fibrinogen concentration in samples of tail vein blood at the time of tumour cell injection and again 12 h later. Fibrinogen was measured by the method of Clauss (1957), on 0.3 ml plasma samples. Thrombotest and fibrinogen values in different groups were compared where appropriate using the *t* test.

*Analysis of procoagulant activity of tumour cells*

Mtln3 cells were trypsinised and washed in F10/DMEM as described above, resuspended in F10/DMEM (without FCS), counted and adjusted to a cell density of $8 \times 10^6$ ml. This suspension (0.1 ml) was added to 0.1 ml of citrated plasma and 0.1 ml of $CaCl_2$ (0.25 M) at 37°C and the clotting time of the mixture recorded electronically using a Dade fibrometer. The procedure was carried out in triplicate with normal human plasma and with bovine plasma deficient in factor VII (factor VII DBP) and in both factors VII and X (factor VII and XDBP). Mtln3 cells were compared with two non-neoplastic cell lines. 3T3 (untransformed mouse fibroblast) cells and MDCK (neonatal dog kidney) cells, and with Mtln3 cells exposed for 72 h *in vitro* to a 10 $\mu$M concentration of warfarin.

### Results

*Experiment 1: Enhancement of metastasis by coagulation factors*

The median number of pulmonary seedlings and semi-interquartile range are recorded for each group in Table I. In the control group (group A), the median number of seedlings per animal was 13, and this was not significantly affected by treatment with factor VII alone (group C, median 11) or with bovine serum albumin (group E, median 23). Treatment with the entire factor complex, or with the component comprising factors II, IX and X produced many more seedlings; the median was 182 in group B and 181 in group D. These results were both very significantly greater than

**Table I** Experiment 1: Number of pulmonary tumour seedlings per animal

| | Group A | Group B | Group C | Group D | Group E |
|---|---|---|---|---|---|
| Median | 13 | 182[a] | 11 | 181[a] | 23 |
| Semi-interquartile range | 5–15 | 84–>200 | 7–25 | 138–>200 | 13–26 |

Accurate estimes of tumour numbers were not attempted in animals with over 200 lung seedlings. [a]Indicates $P<0.001$ on comparison of group results with control group by Mann–Whitney U test.

that for control animals ($P<0.001$), but were not significantly different from each other.

*Experiment 2: Role of fibrin formation in enhancement of metastasis*

The median number of pulmonary seedlings, and the semi-interquartile range for each group are given in Table II. In the control group (group A), the median was 15 tumour deposits per animal; the result for animals treated with arvin alone (group D) was 21 tumours per animal, which was not significantly different from this. As in experiment 1, injection of coagulation factors II, VII, IX and X (group B) enhanced metastasis very markedly, in this case to 157 tumours per animal. Group C animals, which received both arvin and coagulation factor treatment, also showed a very marked enhancement of tumour seeding, to 170 tumours per animal. Both group B and group C, therefore, show a highly significant enhancement of metastasis when compared with the control group ($P<0.01$). The difference between groups B and C themselves was not significant ($P<0.92$).

*Monitoring of coagulation system activity*

The mean thrombotest time for untreated rats in this experiment was 28 sec (range 26–31); rats treated with coagulation factors had a mean of 25 sec (range 25–26), and those treated with arvin a mean of 30.7 sec (range 29–32). Neither of these results was significantly different from that of normal rats. Rats treated with both arvin and coagulation factors (group C) had a mean thrombotest time of 28.5 sec (range 27–30). Arvin, therefore, did not significantly lengthen the thrombotest time, nor did coagulation factor injections significantly shorten it. The mean plasma fibrinogen concentration in control animals was 228.7 mg dl$^{-1}$ (s.d. 16.5 mg dl$^{-1}$). In arvin treated animals the mean value was 76.8 mg dl$^{-1}$ (s.d. 18.8 mg dl$^{-1}$) at the time of tumour cell injection, rising to 116.3 mg dl$^{-1}$ 12 h later (s.d. 20.2). Arvin was therefore effective in reducing the plasma fibrinogen concentration to one-third of the normal at tumour cell injection, and in maintaining a substantial reduction for at least 12 h thereafter.

*Procoagulant activity of tumour cells*

All three cell lines promoted coagulation in this assay. The clotting time in the presence of Mtln3 cells was significantly prolonged in factor VII DPB compared with normal plasma, whilst no clotting occurred in factors VII and X DBP. Similar results were recorded for MDCK and 3T3 cells. Preincubation of Mtln3 cells in 10 $\mu$M warfarin did not affect their ability to promote coagulation of normal or factor deficient plasmas (Table III).

**Table II** Experiment 2: Number of pulmonary tumour seedlings per animal

| | Group A | Group B | Group C | Group D |
|---|---|---|---|---|
| Median | 15 | 157[a] | 170[a] | 21 |
| Semi-interquartile range | 9–27 | 106–198 | 75– >200 | 13–47 |

One animal in group B and two animals in group C died from the effects of repeated ether anaesthesia. The results in these two groups are therefore calculated on 9 and 8 animals respectively. [a]Indicates $P<0.01$ (Mann–Whitney U test for group results against control group).

**Table III** Procoagulant activity of Mtln3, MDCK and 3T3 cells

| | Factor VII DBP | Factor VII and X DBP |
|---|---|---|
| Mtln3 | 147 | >500 |
| Mtln3 (warfarin treated) | 133 | >500 |
| MDCK | 182 | 294 |
| 3T3 | 152 | 367 |

Activities are expressed as a percentage of the clotting time obtained using pooled normal human plasma. Results represent the mean of triplicate assays performed on at least two occasions. Within assay variation was <10%.

### Discussion

The experiments described in this paper demonstrate contrasting effects on metastasis of two different manipulations of the coagulation system. In order to interpret the results correctly, it is important to understand clearly the effect on coagulation of the treatments used.

The injection of an excess of coagulation factors into animals whose coagulation was already optimal did not appear to produce excessive or supranormal coagulation, as measured by thrombotest estimation. This result demonstrated that the preparations used had no significant content of activated factors. It is therefore unlikely that the striking effect of factor complex injections on metastasis can be explained by a major effect on the activity of the coagulation system.

Arvin removes fibrinogen from the circulation by cleaving fibrinopeptide A from the molecule; this produces an inactive monomer, des-A-fibrinogen, which is rapidly cleared by the reticuloendothelial and fibrinolytic systems (Bell *et al.*, 1978). Arvin effectively reduced plasma fibrinogen concentration of our animals by nearly 70% at the time of tumour cell injection. This is roughly equivalent to a reduction of fibrin forming capacity of the same amount. The dose used was chosen to avoid causing any significant circulatory disturbance. Preliminary experiments showed that rats could tolerate very high doses of arvin, but that above 300 IU kg$^{-1}$, significant toxic effects could be observed during i.v. injection. Half of this dose was therefore used in the present experiment. The level of defibrination achieved was similar to the target range in humans when the drug is used clinically as an anticoagulant.

The results of our two experiments can now be summarised. First, injection of the coagulation factors II, VII, IX and X into normal rats greatly enhances the metastasis of tumour cells injected at the same time; second, this effect persists if factor VII is omitted from the complex, whilst this factor alone has no discernible effect on metastasis. Third, a very significant reduction in fibrin forming capacity has no discernible effect on metastasis. Finally, this degree of reduction in fibrin forming capacity does not diminish the enhancing effect on metastasis of the II, VII, IX and X factor complex. These findings indicate that some component of the II, IX and X complex is capable of greatly enhancing metastasis. They also suggest that this enhancing effect may not be dependent on the formation of fibrin. These conclusions are consistent with the results of our previous studies, and with reports (Hagmar, 1972; Donati *et al.*, 1978), that defibrinating agents have no consistent effect on metastasis in other models.

Complete defibrination cannot be achieved with arvin in this model, and it is therefore possible that enough fibrin remains after arvin treatment to fulfil a vital role in enhancing metastasis. However the very marked reduction in metastasis achieved with coumarin anticoagulant treatment (Ryan *et al.*, 1969; Brown, 1973; Hilgard *et al.*, 1978; Poggi *et al.*, 1978; McCulloch & George, 1987) is in striking contrast with the complete absence of any such reduction following arvin treatment. If the formation of a fibrin clot is essential to the metastasis-enhancing process, it is surprising that two major suppressive influences on coagulation should have such contrasting results. Conversely, the marked enhancement of metastasis achieved in normal animals by injecting coagulation factors is unlikely to be due to enhanced coagulation activity, since this could not be detected. Further work is required to confirm our findings, but the evidence of these studies is in favour of a potentiating mechanism which involves specific coagulation factors, rather than coagulation as a whole.

Certain tumour cells have been shown to produce a vitamin K dependent cysteine protease procoagulant (CP) which directly activates factor X (Gordon *et al.*, 1975), and which may be implicated in the process of metastasis (Colucci *et al.*, 1983). Such a molecule might be activated by injections containing factor X, and a modification of the method of Gordon was therefore used to measure the procoagulant capacity of cultured Mtln3 cells. We have previously shown that warfarin treatment of Mtln3 cells prior to injection into a host animal does not affect their metastatic behaviour, and this makes it unlikely that CP plays a major role in the metastatic process in this model. Direct *in vitro* analysis of the procoagulant properties of Mtln3 cells shows that the component of total procoagulant activity which appears to be dependent on factor X (but not factor VII) is not predominant, and is unaffected by warfarin treatment of the cells. These results are similar to those obtained using non-malignant cell lines, and suggest that Mtln3 contains little or no CP.

Our previous results, and those of others (Brown, 1973; McCulloch & George, 1987), suggest that the antimetastatic effect of warfarin probably occurs within the first few hours after tumour cells enter the bloodstream. The model we have adopted allows this part of the metastatic process to be studied closely, whilst eliminating the influence of changes in the primary tumour: It has also enabled us to design useful experiments which would not have been possible in a more complete model of metastasis. The use of a small number of cells from a genuinely metastatic neoplasm minimised the risk, which arises in such models (Poggi *et al.*, 1981) of artefacts caused by effects of the cell injection on the coagulation and immune systems, and on the lung vasculature.

The mechanism by which the factors II, IX and X complex enhances metastasis cannot be deduced from this work. Interactions between certain coagulation factors, notably factor XII and other biological systems such as the complement system, the kinin system, fibrinolysis and platelet activation are known to occur (Zimmerman *et al.*, 1977), and the metastasis-enhancing effect may be mediated via a similar mechanism. Our findings must cast doubt on many of the commonly proposed theories for the interaction of cancer with coagulation, which have assumed that the interaction occurs at the level of fibrin clot formation (Zacharski, 1984). If confirmed by further studies, these results will require the formulation of quite different theories of the cancer/coagulation relationship. The finding that intravenous injection of coagulation factors enhances metastasis may, if it can be shown to extend to the human situation, have implications for the current controversy on the effects of perioperative blood transfusion on survival in cancer patients.

We would like to thank Drs Susan North and Garth Nicolson of the M.D. Anderson Tumor Institute for the generous gift of a culture of Mtln3 cells; Drs J.K. Smith and R.J. Perry of the Blood Transfusion Service for their gifts of factor VII and factors II, IX and X concentrates respectively: and Dr Jane Plumb for her helpful criticism. Thanks also to Mr Colin Hughes for his assistance and to Yvonne Galbraith for typing the manuscript. Part of this work was completed at the CRC Department of Oncology, University of Glasgow, and we would like to thank Dr Ian Freshney for allowing us to use these facilities, and for the gift of the cell lines MDCK and 3T3.

### References

AGOSTINO, D., CLIFFTON, E.E. & GIROLAMI, A. (1966). Effect of prolonged coumadin treatment on the production of pulmonary metastasis in the rat. *Cancer*, **19**, 284.

BELL, W.R., SHAPIRO, S.S., MARTINEZ, J. & NOSSEL, H.L. (1978). The effects of Ancrod, the coagulation inhibitor of the Malayan pit viper (A Rhodostoma) on prothrombin and fibrinogen metabolism and fibrinopeptide A release in man. *J. Lab. Clin. Med.*, **91**, 592.

BROWN, J.M. (1973). A study of the mechanism by which anticoagulation with warfarin inhibits blood-borne metastases. *Cancer Res.*, **33**, 1217.

CLAUSS, A. (1957). Gerrinungsphysiologische schnell methode zur bestimmung des fibrinogens. *Acta Haematol.*, **17**, 237.

COLUCCI, M., DELAINI, F., DE BELLIS VITI, G. & 4 others (1983). Warfarin inhibits both procoagulant activity and metastatic capacity of Lewis lung carcinoma cells. *Biochem. Pharmacol.*, **32**, 1689.

DONAT, M.B., MUSSONI, L., POGGI, A., DE GAETANO, G. & GARATTINI, S. (1978). Growth and metastasis of the Lewis lung carcinoma in mice defibrinated with batroxobin. *Eur. J. Cancer*, **14**, 343.

DVORAK, H.F., DICKERSIN, G.R., DOVORAK, A.M., MANSEAU, E.J. & PYNE, K. (1981). Human breast carcinoma: Fibrin deposits and desmoplasia, inflammatory cell type and distribution: Microvasculature and infarction. *J. Natl Cancer Inst.*, **67**, 335.

GORDON, S.G., FRANKS, J.J. & LEWIS, B. (1975). Cancer procoagulant A: A factor X activating procoagulant from malignant tissue. *Thromb. Res.*, **6**, 127.

HAGMAR, B. (1972). Defibrination and metastasis formation: Effects of arvin on experimental metastasis in mice. *Eur. J. Cancer*, **8**, 17.

HILGARD, P., SCHULTE, H., WETZIG, G., SCMITT, G. & SCHMIDT, C.G. (1977). Oral anticoagulation in the treatment of spontaneously metastasising murine tumour (3LL). *Br. J. Cancer*, **35**, 78.

HILGARD, P. & MAAT, B. (1979). Mechanism of lung tumour colony reduction caused by coumarin anticoagulation. *Eur. J. Cancer*, **15**, 183.

KOIKE, A. (1964). Mechanism of blood-borne metastasis. *Cancer*, **17**, 450.

MANNUCI, P.M., VAGLINI, M., MANIEZZO, M., E. MAGNI, D. MARI & N. CASCINELLI (1985). Haemostatic alterations are unrelated to stage of tumour in untreated malignant melanoma and breast cancer. *Eur. J. Cancer Clin. Oncol.*, **21**, 681.

McCULLOCH, P.G. & GEORGE, W.D. (1987). Warfarin inhibition of metastasis; the role of anticoagulation. *Br. J. Surg.*, **74**, 879.

NERI, A. & NICOLSON, G.L. (1981). Phenotypic drift of metastatic and cell surface properties of mammary adenocarcinoma cell clones during growth *in vitro*. *Int. J. Cancer.*, **28**, 731.

NERI, A., WELCH, D., KAWAGUCHI, T. & NICOLSON, G.L. (1982). The development and biological properties of malignant cell sublines and clones of a spontaneously metastasising rat mammary carcinoma. *J. Natl Cancer Inst.*, **68**, 507.

O'MEARA, R.A.Q. (1968). Fibrin formation and tumour growth. *Thromb. Diath. Haemorrh. Supp.*, **28**, 137.

POGGI, A., MUSSONI, L. KORNBLUHT, L., E. BALLABIO, G. DE GAETANO & M. B. DONATI (1978). Warfarin enantiomers, anticoagulation and experimental tumour metastasis. *Lancet.* **i**, 163.

POGGI, A. DONATI, M.B. & GARATTINI, S. (1981). Fibrin and cancer cell growth; problems in the evaluation of experimental models. In *Malignancy and the Haemostatic System*, M.B. Donati *et al.* (eds) p. 89. Raven: New York.

RICKLES, F.R. & EDWARDS, R.L. (1983). Activation of blood coagulation in cancer; Trousseau's syndrome revisited. *Blood*, **62**, 14.

RYAN, J.J., KETCHAM, A.S. & WEXLER, H. (1969). Warfarin therapy as an adjunct to the surgical treatment of malignant tumours in mice. *Cancer Res.*, **29**, 2191.

SEGALOFF, A. (1966). Hormones and breast cancer. *Rec. Prog. Hormone Res.*, **22**, 351.

STENFLO, J. & SUTTIE, J.W. (1977). Vitamin K dependent formation of gamma-carboxyglutamic acid. *Ann. Rev. Biochem.*, **46**, 157.

SUN, N.C.J., McAFEE, W.M., HUM, G.J. & WEINER, J.M. (1979). Haemostatic abnormalities in malignancy; a prospective study of 108 patients. *Am. J. Clin. Path.*, **71**, 10.

WEXLER, H. (1966). Accurate identification of experimental pulmonary metastases. *J. Natl Cancer Inst.*, **36**, 641.

WILLIAMSON, R.C.N., LYNDON, P.J. & TUDWAY, A.J.C. (1980). The effects of anticoagulation and ileal resection on the development and spread of experimental intestinal carcinomas. *Br. J. Cancer*, **42**, 85.

WOOD, S., JR. (1958). Pathogenesis of metastasis formation observed *in vivo* in the rabbit ear chamber. *Arch. Pathol.*, **66**, 550.

WOOD, S., JR. (1974). Experimental studies on the spread of cancer with respect to fibrinolytic agents and anticoagulants. *J. Med.*, **5**, 7.

ZACHARSKI, L.R., HENDERSON, W.G., RICKLES, F.R. & 5 others (1979). Rationale and experimental design for the VA cooperative study of anticoagulation (warfarin) in the treatment of cancer. *Cancer*, **44**, 732.

ZACHARSKI, L.R. (1984). Rationale for anticoagulant treatment of cancer. In *Haemostatic Mechanisms and Metastasis*, K.V. Honn and B.F. Sloane (eds) Ch. 24, p. 368. Martinus Nijhoff: Boston.

ZIMMERMAN, T.S., FIERER, J. & ROTHBERGER, H. (1977). Blood coagulation and the inflammatory response. *Semin. Haematol*, **14**, 391.


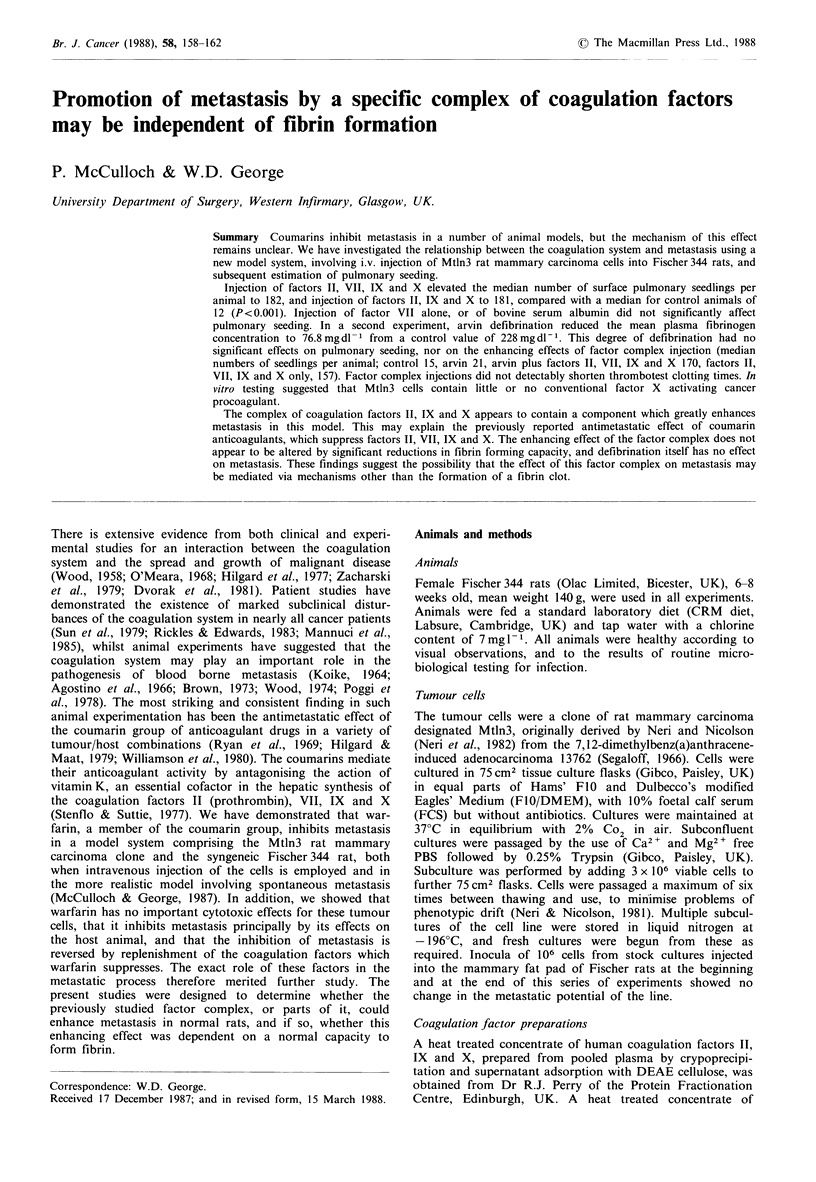

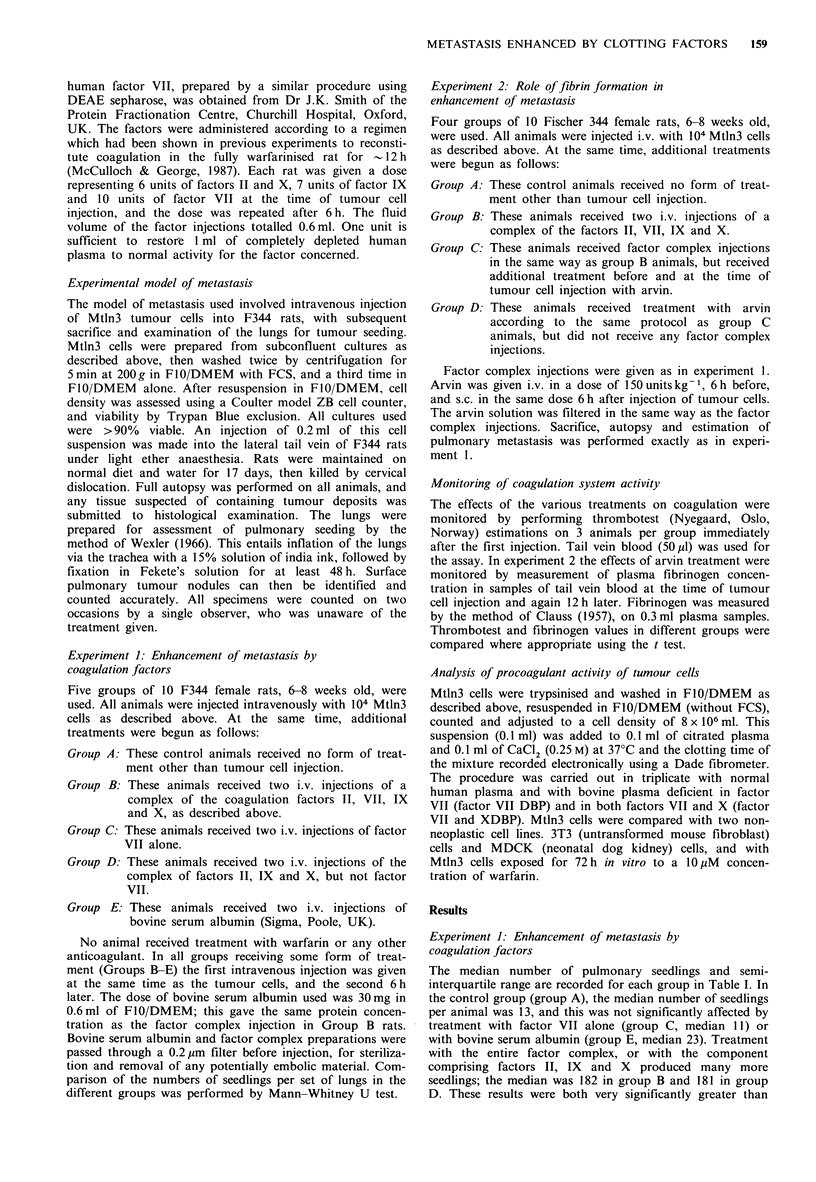

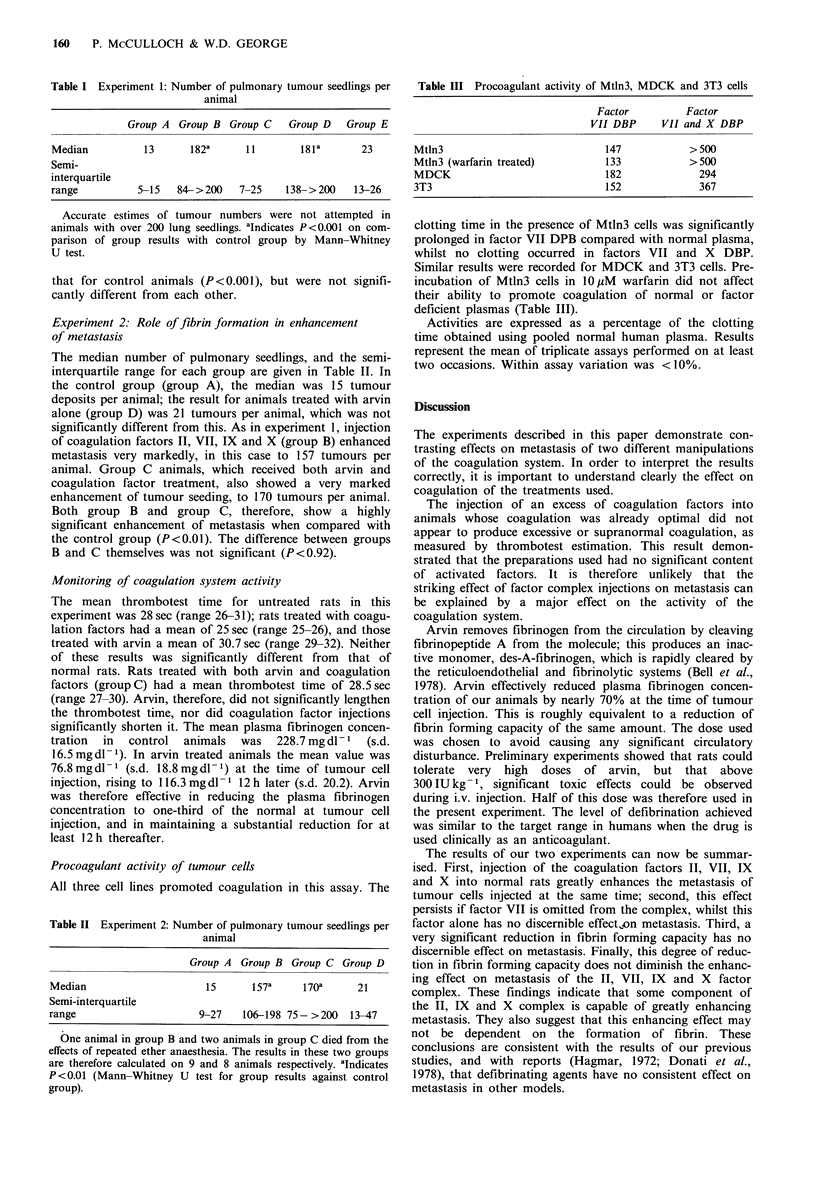

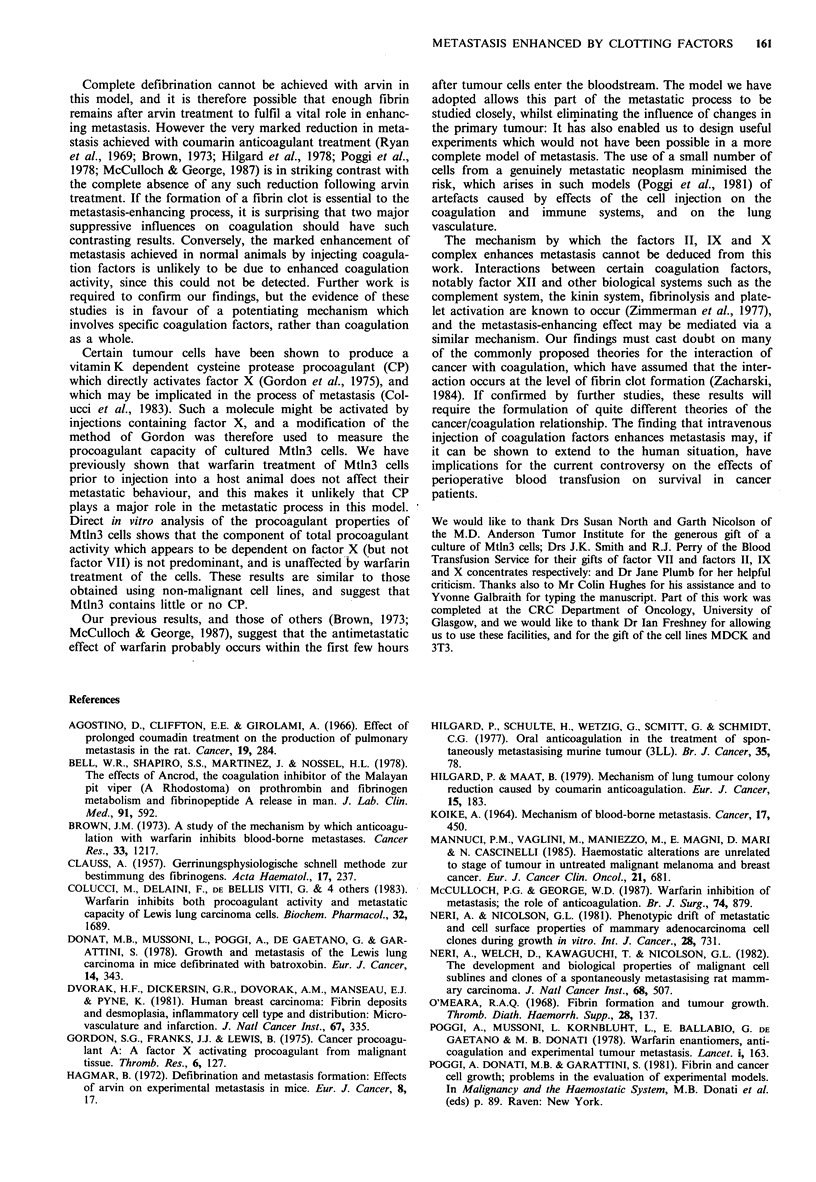

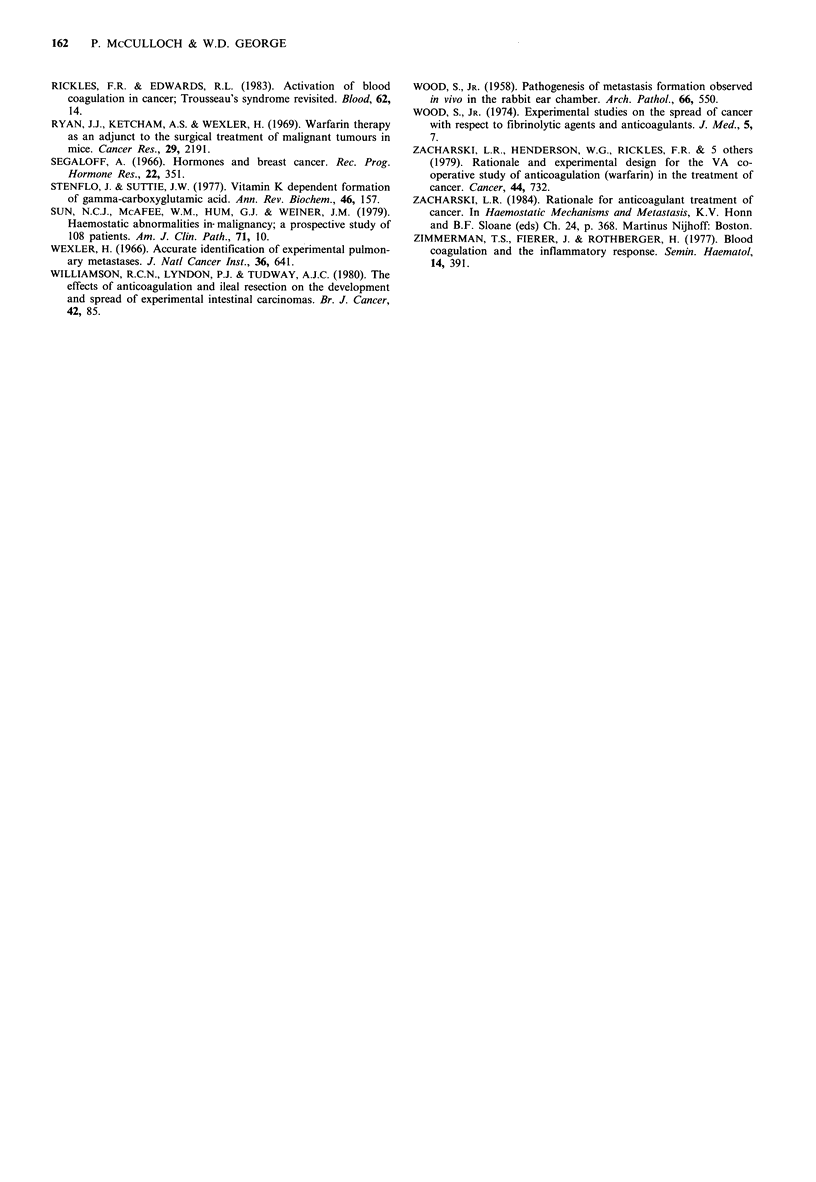


## References

[OCR_00543] Agostino D., Cliffton E. E., Girolami A. (1966). Effect of prolonged coumadin treatment on the production of pulmonary metastases in the rat.. Cancer.

[OCR_00548] Bell W. R., Shapiro S. S., Martinez J., Nossel H. L. (1978). The effects of ancrod, the coagulating enzyme from the venom of Malayan pit viper (A. rhodostoma) on prothrombin and fibrinogen metabolism and fibrinopeptide A release in man.. J Lab Clin Med.

[OCR_00555] Brown J. M. (1973). A study of the mechanism by which anticoagulation with warfarin inhibits blood-borne metastases.. Cancer Res.

[OCR_00560] CLAUSS A. (1957). Gerinnungsphysiologische Schnellmethode zur Bestimmung des Fibrinogens.. Acta Haematol.

[OCR_00564] Colucci M., Delaini F., de Bellis Vitti G., Locati D., Poggi A., Semeraro N., Donati M. B. (1983). Warfarin inhibits both procoagulant activity and metastatic capacity of Lewis lung carcinoma cells. Role of vitamin K deficiency.. Biochem Pharmacol.

[OCR_00572] Donati M. B., Mussoni L., Poggi A., De Gaetano G., Garattini S. (1978). Growth and metastasis of the Lewis lung carcinoma in mice defibrinated with batroxobin.. Eur J Cancer.

[OCR_00576] Dvorak H. F., Dickersin G. R., Dvorak A. M., Manseau E. J., Pyne K. (1981). Human breast carcinoma: fibrin deposits and desmoplasia. Inflammatory cell type and distribution. Microvasculature and infarction.. J Natl Cancer Inst.

[OCR_00582] Gordon S. G., Franks J. J., Lewis B. (1975). Cancer procoagulant A: a factor X activating procoagulant from malignant tissue.. Thromb Res.

[OCR_00587] Hagmar B. (1972). Defibrination and metastasis formation: effects of arvin on experimental metastases in mice.. Eur J Cancer.

[OCR_00598] Hilgard P., Maat B. (1979). Mechanism of lung tumour colony reduction caused by coumarin anticoagulation.. Eur J Cancer.

[OCR_00592] Hilgard P., Schulte H., Wetzig G., Schmitt G., Schmidt C. G. (1977). Oral anticoagulation in the treatment of a spontaneously metastasising murine tumour (3LL).. Br J Cancer.

[OCR_00603] KOIKE A. (1964). MECHANISM OF BLOOD-BORNE METASTASES. I. SOME FACTORS AFFECTING LODGMENT AND GROWTH OF TUMOR CELLS IN THE LUNGS.. Cancer.

[OCR_00607] Mannucci P. M., Vaglini M., Maniezzo M., Magni E., Mari D., Cascinelli N. (1985). Hemostatic alterations are unrelated to the stage of tumor in untreated malignant melanoma and breast carcinoma.. Eur J Cancer Clin Oncol.

[OCR_00613] McCulloch P., George W. D. (1987). Warfarin inhibition of metastasis: the role of anticoagulation.. Br J Surg.

[OCR_00617] Neri A., Nicolson G. L. (1981). Phenotypic drift of metastatic and cell-surface properties of mammary adenocarcinoma cell clones during growth in vitro.. Int J Cancer.

[OCR_00622] Neri A., Welch D., Kawaguchi T., Nicolson G. L. (1982). Development and biologic properties of malignant cell sublines and clones of a spontaneously metastasizing rat mammary adenocarcinoma.. J Natl Cancer Inst.

[OCR_00628] O'Meara R. A. (1968). Fibrin formation and tumour growth.. Thromb Diath Haemorrh Suppl.

[OCR_00632] Poggi A., Mussoni L., Kornblihtt L., Ballabio E., de Gaetano G., Donati M. B. (1978). Warfarin enantiomers, anticoagulation, and experimental tumour metastasis.. Lancet.

[OCR_00644] Rickles F. R., Edwards R. L. (1983). Activation of blood coagulation in cancer: Trousseau's syndrome revisited.. Blood.

[OCR_00649] Ryan J. J., Ketcham A. S., Wexler H. (1969). Warfarin therapy as an adjunct to the surgical treatment of malignant tumors in mice.. Cancer Res.

[OCR_00654] Segaloff A. (1966). Hormones and breast cancer.. Recent Prog Horm Res.

[OCR_00658] Stenflo J., Suttie J. W. (1977). Vitamin K-dependent formation of gamma-carboxyglutamic acid.. Annu Rev Biochem.

[OCR_00662] Sun N. C., McAfee W. M., Hum G. J., Weiner J. M. (1979). Hemostatic abnormalities in malignancy, a prospective study of one hundred eight patients. Part I. Coagulation studies.. Am J Clin Pathol.

[OCR_00677] WOOD S. (1958). Pathogenesis of metastasis formation observed in vivo in the rabbit ear chamber.. AMA Arch Pathol.

[OCR_00667] Wexler H. (1966). Accurate identification of experimental pulmonary metastases.. J Natl Cancer Inst.

[OCR_00671] Williamson R. C., Lyndon P. J., Tudway A. J. (1980). Effects of anticoagulation and ileal resection on the development and spread of experimental intestinal carcinomas.. Br J Cancer.

[OCR_00681] Wood S. (1974). Experimental studies on the spread of cancer, with special reference to fibrinolytic agents and anticoagulants.. J Med.

[OCR_00686] Zacharski L. R., Henderson W. G., Rickles F. R., Forman W. B., Cornell C. J., Forcier R. J., Harrower H. W., Johnson R. O. (1979). Rationale and experimental design for the VA Cooperative Study of Anticoagulation (Warfarin) in the Treatment of Cancer.. Cancer.

[OCR_00696] Zimmerman T. S., Fierer J., Rothberger H. (1977). Blood coagulation and the inflammatory response.. Semin Hematol.

